# Effect of 5-HT2A receptor antagonism on levels of D2/3 receptor occupancy and adverse behavioral side-effects induced by haloperidol: a SPECT imaging study in the rat

**DOI:** 10.1038/s41398-020-01179-5

**Published:** 2021-01-14

**Authors:** Stergios Tsartsalis, Benjamin B. Tournier, Yesica Gloria, Philippe Millet, Nathalie Ginovart

**Affiliations:** 1 Division of Adult Psychiatry, Department of Psychiatry, Geneva University Hospitals, Geneva, Switzerland; 2 Division of Psychiatric Specialties, Department of Psychiatry, Geneva University Hospitals, Geneva, Switzerland; 3 Department of Psychiatry, Faculty of Medicine, University of Geneva, Geneva, Switzerland; 4 Department of Basic Neurosciences, Faculty of Medicine, University of Geneva, Geneva, Switzerland

**Keywords:** Neuroscience, Pharmacology, Schizophrenia

## Abstract

Several studies suggested that 5-HT_2A_ receptor (5-HT_2A_R) blockade may provide a more favorable efficacy and side-effect profile to antipsychotic treatment. We hypothesized that a combined haloperidol (a D_2/3_ receptor (D_2/3_R) antagonist) and MDL-100,907 (a 5-HT_2A_R antagonist) treatment would reverse the side effects and the neurochemical alterations induced by haloperidol alone and would potentialize its efficacy. We thus chronically treated male Mdr1a knock-out rats with several doses of haloperidol alone or in combination with a saturating dose of a MDL-100,907. Receptor occupancy at clinically relevant levels was validated with a dual-radiotracer in-vivo SPECT imaging of D_2/3_R and 5-HT_2A_R occupancy. Experimental tests of efficacy (dizocilpine-disrupted prepulse inhibition (PPI) of the startle reflex) and side effects (catalepsy, vacuous chewing movements) were performed. Finally, a second dual-radiotracer in-vivo SPECT scan assessed the neurochemical changes induced by the chronic treatments. Chronic haloperidol failed to reverse PPI disruption induced by dizocilpine, whilst administration of MDL-100,907 along with haloperidol was associated with a reversal of the effect of dizocilpine. Haloperidol at 0.5 mg/kg/day and at 1 mg/kg/day induced catalepsy that was significantly alleviated (by ~50%) by co-treatment with MDL-100,907 but only at 0.5 mg/kg/day dose of haloperidol. Chronic haloperidol treatment, event at doses as low as 0.1 mg/kg/day induced a significant upregulation of the D_2/3_R in the striatum (by over 40% in the nucleus accumbens and over 20% in the caudate-putamen nuclei), that was not reversed by MDL-100,907. Finally, an upregulation of 5-HT_2A_R after chronic haloperidol treatment at a moderate dose only (0.25 mg/kg/day) was demonstrated in frontal cortical regions and the ventral tegmental area. Overall, a partial contribution of a 5-HT_2A_R antagonism to the efficacy and side-effect profile of antipsychotic agents is suggested.

## Introduction

Antipsychotic medication constitutes the cornerstone of schizophrenia treatment. Antipsychotic agents are classified into typical (mainly D_2_ receptor, D_2_R, antagonists with relatively low affinity for other receptors) and atypical (with affinity for a wide spectrum of receptors, apart from the D_2_R) (reviewed in ref. ^1^). D_2_R antagonism is a central element of antipsychotic activity^2^. Indeed, for the majority of antipsychotic agents, a D_2_R occupancy between 65% and 80% of the total receptor pool in the striatum is associated with optimal antipsychotic efficacy. An occupancy below this level produces no antipsychotic effect, whereas a higher occupancy is associated with the appearance of—mainly—extrapyramidal side effects (EPS)^1^.

When compared to typical agents, atypical antipsychotics possess a lower propensity to cause EPS^3,4^. This suggests that a better understanding of the mechanism of action of atypical antipsychotics could lead to the design of a more tolerable, hence, more efficient treatment of schizophrenia. Despite extensive efforts, the neurochemical and/or molecular bases of atypicality have long been a matter of debate. One popular theory proposes that a high 5-HT_2A_ vs. D_2_R occupancy is a defining characteristic of atypical antipsychotics and indeed, the majority of them has a high affinity for the 5-HT_2A_ receptor (5-HT_2A_R)^5^. Whereas 5-HT_2A_R antagonism per se is not considered as conferring antipsychotic efficacy^6^, a combined blockade of D_2_ and 5-HT_2A_R has been proposed to be important for the efficacy and the reduced side effect liability of atypical versus typical drugs^1,2,7^. The existing literature in the field is controversial and a systematic approach to the question of the implication of a 5-HT_2A_R antagonism in antipsychotic atypicality is needed. Indeed, many studies have assessed the effect of 5-HT_2A_R antagonism in association with D_2_R blockade, notably by haloperidol, on a wide spectrum of behavioral paradigms of antipsychotic efficacy and side effect liability in rodents. However, in most studies, a single and, in most cases, saturating dose of haloperidol has been used^7–12^. In addition, to our knowledge, no study has simultaneously assessed multiple aspects of antipsychotic efficacy and side effect profile.

In the present study, we chronically treated male rats with several doses of haloperidol alone or in combination with a saturating dose of a selective 5-HT_2A_R antagonist, MDL-100,907. The occupancy of D_2/3_R at clinically relevant levels, from subtherapeutic doses occupying <65% of the D_2/3_R in the striatum, to doses within the optimal therapeutic “window” of D_2_R occupancy (65–80%) and saturating, supratherapeutic doses (frequently associated to EPS), was validated using a dual-radiotracer single-photon emission computed tomography (SPECT) imaging approach to assess D_2/3_R and 5-HT_2A_R occupancies, simultaneously, during the same scan session^13^. In parallel, the effects of adding 5-HT_2A_ to different levels of D_2/3_R occupancies were investigated using a series of preclinical tests of efficacy (dizocilpine—also known as MK801—disrupted prepulse inhibition (PPI) of the startle reflex) and side effects (catalepsy, vacuous chewing movements (VCM)). Finally, a second dual-radiotracer in-vivo SPECT scan was performed following a 4-week treatment period to assess neurochemical changes at the level of D_2/3_R and 5-HT_2A_R binding with respect to the chronic treatment regimes. Our hypothesis was that adding 5-HT_2A_R antagonism, a putative substrate of antipsychotic atypicality, could enhance the efficacy of haloperidol at the experimental tasks and alleviate, at least partially, EPS.

## Materials and methods

### Animals

A total of 136 male adult Mdr1a knock-out rats (weighing 300–500 g), were used. P-glycoprotein knock-out in this strain increases the permeability of the blood–brain barrier, allowing in-vivo 5-HT_2A_R imaging with [^125^I]R91150, which is impeded in wild-type animals due to the low brain absorption of this radiotracer^13–15^. The animals were housed at constant room temperature (21 ± 1 °C) under a regular light/dark schedule (light 07:00–19:00). Food and water were freely available.

All experimental procedures were performed in accordance with the Swiss Federal Law and approved by the local authority on Animal Experimentation.

### Experimental procedures outline

**Fig. 1:**
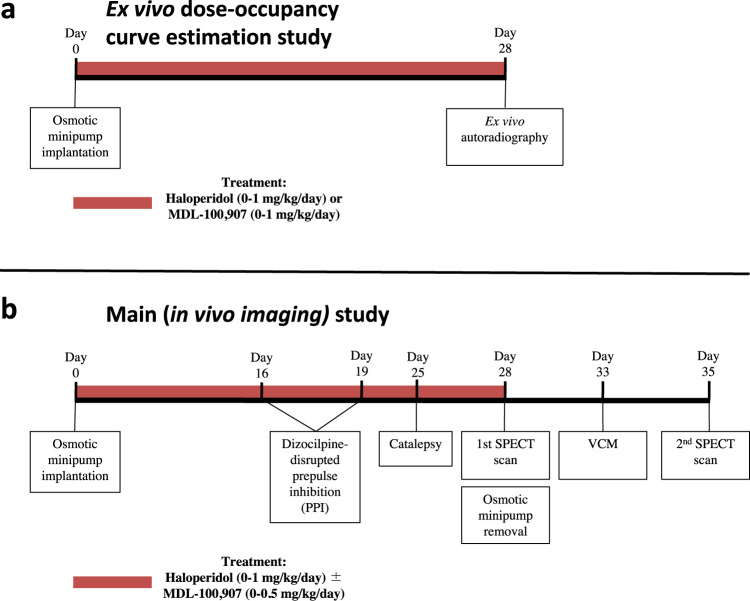
Graphical presentation of the timeline of the present study. The black line represents the timeline of the experiments with the various time-points (in days) noted above the line. The red line represents the period during which the animals were under treatment. The square boxes below the black line describe the behavioral and/or imaging experiments undetaken at specific time-points.

The timeline of the study is graphically presented in Fig. 1. An initial ex-vivo study was performed to determine the dose-occupancy of haloperidol and MDL-100,907 at D_2/3_R and 5-HT_2A_R, respectively, in our model. Using subcutaneously implanted osmotic minipumps, ranging doses of haloperidol and MDL-100,907 were administered (in separate groups of rats) to produce chronic and stable levels of D_2/3_R and 5-HT_2A_R occupancies. At the end of a 4-week treatment period, ex-vivo receptor binding measurements were performed to establish the dose-occupancy curve for haloperidol and MDL-100,907. Based on the ex-vivo D_2/3_R occupancy results, doses of haloperidol that achieved: (1) subtherapeutic/at the lower spectrum of the optimal occupancy window, (2) therapeutic levels of D_2_R occupancy (within the 65–80% occupancy window) and, (3) supratherapeutic doses associated to a side effect risk (>80% of occupancy)^1^, were selected and used alone or in combination with a 5-HT_2A_R saturating dose of MDL-100,907. This allowed to compare, in vivo, their chronic effects both on behavioral tasks designed to assess clinical efficacy and EPS liability and on D_2/3_R and 5-HT_2A_R binding.

### Osmotic minipump implantation procedure and chronic drug treatment

Haloperidol and MDL-100,907 (Sigma-Aldrich, Buchs, Switzerland) were diluted in a 50% DMSO solution in NaCl 0.9% (50% v/v). MDL-100,907 was initially diluted in a few drops of a 10% acetic acid solution (constituting <5% of the final volume of the DMSO/NaCl solution). For both haloperidol and MDL-100,907, the doses used in the initial ex-vivo dose-occupancy curve estimation study ranged from 0 to 1 mg/kg/day. The haloperidol/MDL-100,907 doses used in the subsequent in-vivo study (hereon abbreviated as Hx/My with x and y being the dose of each drug in mg/kg/day) and the number of rats (*n*) in each dosage were as follows: H0/M0 (*n* = 12), H0.1/M0 (*n* = 8), H0.1/M0.5 (subtherapeutic dose of haloperidol) (*n* = 7), H0.25/M0 (*n* = 7), H0.25/M0.5 (*n* = 7) (therapeutic dose), H0.5/M0 (*n* = 7), H0.5/M0.5 (*n* = 7), H1/M0 (*n* = 12), and H1/M0.5 (*n* = 12) (supratherapeutic doses). The doses employed in the in vivo study were informed by the ex vivo dose-occupancy estimation study. The rats were randomly assigned into dose groups by shuffling the rat ID/dose labels. The investigators who performed the experiments were totally blinded to the group and dose assignment of rats. Investigators who analyzed the results were aware of the group-assignment but blinded to the dose assignment to each group. Exclusion criteria included signs of local (at the surgical site) and generalized infection, dehydration, rapid weight loss, and lethargy.

Osmotic minipump (2ML4, Alzet, Cupertino, CA, USA) implantation, localized between the scapulae, was performed under isoflurane anesthesia (2.5–3%) and buprenorphine analgesia (0.02 mg/kg sc; Temgesic, Reckitt Benckiser Pharmaceuticals Inc.). For a more detailed description, please see the supplemental materials and methods.

At the end of the 28 days treatment period, the minipumps were removed to end the chronic administration period.

### Behavioral testing

The dizocilpine-disrupted PPI of the startle reflex was performed as a proxy to the therapeutic efficacy of the chronic treatment^16–18^, between 16 and 19 days following implantation of the osmotic minipumps. The protocol described here^19^ was followed, including two habituation sessions (Days 1 and 2) and a saline-pretreatment test session (Day 3). Dizocilpine (0.15 mg/kg) was injected as pretreatment on Day 4. The amplitude of startle responses was recorded in all trials. The magnitude of PPI was calculated as a percent inhibition of the startle amplitude in the pulse-alone trial^18,20^, using two prepulse sound volumes (80 and 85 dB).

Catalepsy is indicative of the potential of a pharmacological agent to induce extrapyramidal symptoms^1,7^. At 25 days following minipump implantation, catalepsy was assessed over a 3-min period as described previously^21^ (see supplemental Materials and Methods).

VCM are purposeless, vertical jaw movements directed towards no object. They are considered a rodent model of antipsychotic drug-induced tardive dyskinesia^1,7,11,22,23^. The assessment of VCM took place 5 days after the removal of the osmotic minipumps, i.e. the end of the treatment period. To assess VCM, rats were placed in a plexiglass restraining tube. After 2 min of habituation, VCM were recorded over a period of 2 min^24,25^.

### Ex-vivo receptor-binding measurements and in-vivo imaging

#### Ex-vivo estimation of receptor occupancy by haloperidol and MDL-100,907

Preparation of [^123^I]IBZM and [^125^I]R91150 was performed as previously described^13–15^. In the ex-vivo dose-occupancy curve estimations, rats were administered with [^123^I]IBZM and [^125^I]R91150 to concurrently measure D_2/3_R and 5-HT2AR occupancy, respectively. At 28 days of treatment, rats were anesthetized using isoflurane anesthesia (4% for induction, 2.5% for maintenance) and injected with 6.48 ± 0.34 MBq of [^123^I]IBZM or 6.98 ± 0.98 MBq of [^123^I]R91150 (with respect to the treatment, haloperidol or MDL-100,907, respectively). At 120 min post-injection, rats were euthanized by decapitation, their brain removed, and their striatum, frontal cortex, and cerebellum dissected and weighed. Radioactivity in the dissected brain regions was immediately measured in an automated gamma counting system (expressed in kBq/g of tissue weight) for the radiotracer labeled with ^123^I. Radioactivity was decay-corrected to the time of the brain dissection.

For the ex-vivo study, the standardized uptake ratio (SUR) for each radiotracer in the striatum and the frontal cortex was measured using the radioactivity measured in the gamma counting system as follows: SUR = (radioactivity in the target-region)/(radioactivity in the cerebellum)–1. The % occupancy (O) of the D_2/3_R and the 5-HT_2A_R from their respective antagonists was estimated using the following formula: O (%) = (1−SUR/SUR_CON_)*100, where SUR corresponds to the value obtained from an individual study in which a dose of antagonist was employed, while SUR_CON_ corresponds to the average value obtained from the control animals in which no antagonist was administered.

#### In-vivo imaging experiments

Dual-radiotracer SPECT imaging^13^ was performed in the context of the main in-vivo study described in this paper to assess the level of D_2/3_R and 5-HT_2A_R occupancy by haloperidol and MDL-100,907, and the binding of D_2/3_R and 5-HT_2A_R after chronic treatment with these agents. In vivo dual radiotracer SPECT was performed as described previously^13^. At the end of the 28-day treatment period, the first dual-radiotracer SPECT scan was performed, to measure the occupancy of the D_2/3_R and the 5-HT_2A_R by their respective antagonists. One week later, an identical dual-radiotracer SPECT scan was performed to index the density of the D_2/3_R and the 5-HT_2A_R. Rats were simultaneously injected with a mixture of [^123^I]IBZM (32.7 ± 8.2 MBq) and [^125^I]R91150 (26.9 ± 6 MBq) over 30 s. The detailed scan procedures were exactly the same as described here^13,15^.

SPECT image analysis was performed as described previously^13^. A volume-of-interest (VOI) template incorporated in PMOD^26^ was used to extract the radioactivity from each brain VOI and the cerebellum (CER), which was used as reference region. SUR values from the first (to estimate the receptor occupancies by the antagonist treatment) and the second SPECT scan (to estimate the alteration in receptors’ binding due to the chronic treatment) were estimated as follows: (radioactivity in the target VOI)/(radioactivity in CER)−1. For the estimation of D_2/3_R occupancies using in-vivo imaging with [^123^I]IBZM, a 0.55 value was subtracted from the SUR and SUR_CON_ values to account for the difference in the non-displaceable binding between the striatum (target region) and the cerebellum (reference region) for this radiotracer^15^ (please see the supplemental materials and methods for a more detailed description).

### Statistical analysis

Normal distribution of data was assessed using the Shapiro–Wilk test. Post-hoc analysis was performed when appropriate. For the analysis of the PPI, as well as for the analysis of the alterations in D_2/3_ binding, a multi-variate analysis of variance (MANOVA) was employed with haloperidol and MDL-100,907 dose as the independent factors. For non-normally distributed data, non-parametric tests (Kruskal–Wallis and Mann–Whitney) were employed. A sample size analysis with the graphical Douglas Altman’s nomogram was performed^27^. For 5-HT_2A_R binding, parametric images of SUR were compared between groups using the SPM12 software (Wellcome Trust Centre for Neuroimaging, UCL, London, UK) and the Small Animal Molecular Imaging Toolbox^28^ (SAMIT, Groningen, Netherlands) in Matlab (R2019, Mathworks Inc, USA). An uncorrected *p* at 0.001 with a cluster size threshold of 100 voxels was employed^29,30^. All the statistical tests were two-sided. No adjustment for multiple comparisons was employed. Throughout the manuscript, “average” refers to the mean value. All experiments were performed once. All data associated with this manuscript is available upon request to the corresponding author.

## Results

### Occupancy of the D_2/3_R and the 5-HT_2A_R by haloperidol and MDL-100,907

**Fig. 2:**
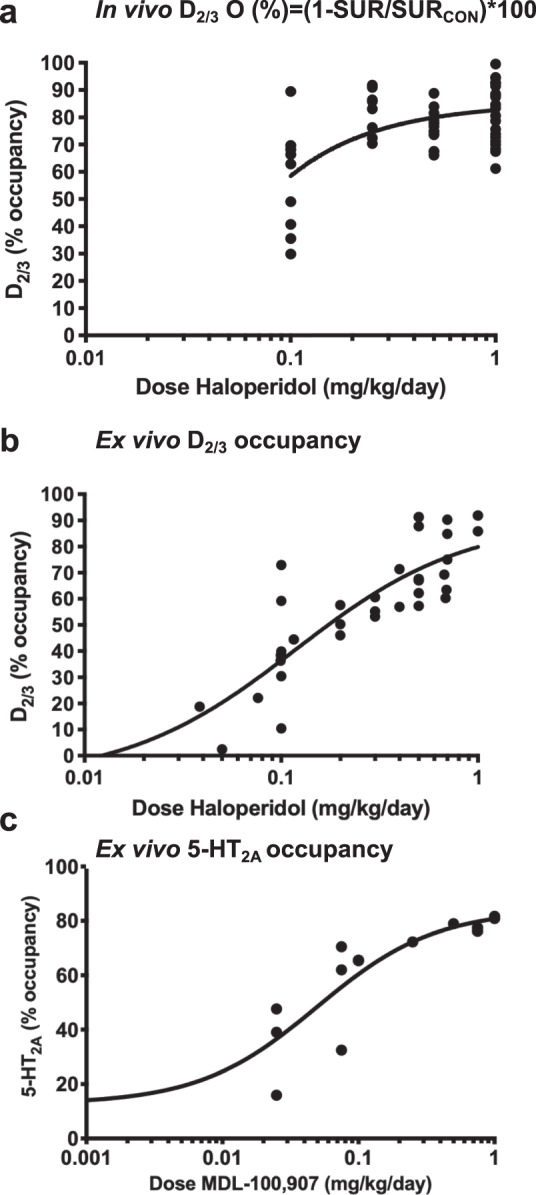
Haloperidol and MDL-100,907 dose-occupancy curves. Fig. 2a and 2b shows the occupancy of the D_2/3_R in the striatum by various doses of haloperidol estimated in vivo and ex vivo, respectively. The equation used to calculate the occupancy is shown above Fig. 2a. Fig. 2c shows the occupancy of 5-HT2AR in the frontal cortex of rats by various doses of MDL-100,907, estimated ex vivo.

Figure 2a, b present the dose-occupancy curves for haloperidol from the in vivo and the initial ex vivo occupancy estimations, respectively. Both in vivo and ex vivo dose-occupancy curve estimation approaches yielded similar results. For haloperidol, a 0.1 mg/kg/day dose leads to a D_2/3_R occupancy of around 45% (Fig. 2a). A dose of 0.25 mg/kg/day leads to a D_2/3_R occupancy of a little <80%, while the doses of 0.5 and 1 mg/kg/day lead towards saturations of more than 85–90% of the D_2/3_R in the Caudate-Putamen (CP; Fig. 2a). The ex-vivo dose-occupancy curve for MDL-100,907 at frontal 5-HT_2A_R is shown in Fig. 2c. The MDL-100,907 dose of 0.5 mg/kg/day, which produces an almost total saturation of the frontal 5-HT_2A_R, was subsequently employed in the in-vivo study. Haloperidol did not induce 5-HT_2A_R occupancy and MDL-100,907 did not induce D_2/3_R occupancy at any dose (data not shown).

### Effect of chronic haloperidol and MDL-100,907 on the dizocilpine-disrupted PPI of the startle

**Fig. 3:**
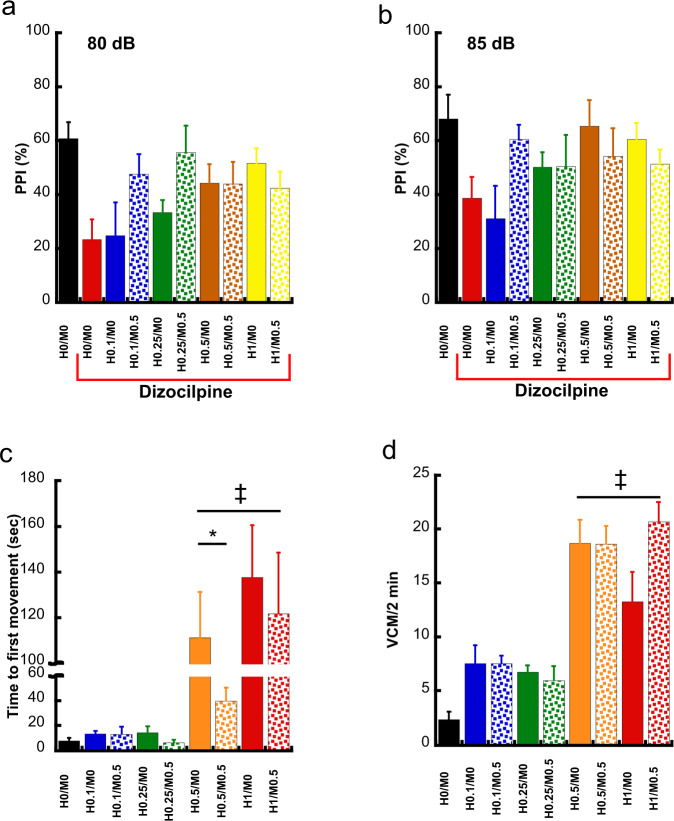
Results of the behavioral tests. The effect of the various haloperidol and MDL-100,907 combinations on the disruption of the PPI by dizocilpine using a (**a**) 80 dB and a (**b**) 85 dB auditory pulse (mean ± SEM values). The leftmost bar corresponds to the control group (H0/M0), not pre-treated with dizocilpine (baseline PPI). The rest correspond to rats pretreated with dizocilpine. The haloperidol and MDL-100,907 dosages are depicted below each bar. No significant differences were found in pairwise comparisons. **c** Results of the catalepsy tests under the various chronic treatment combinations (mean±SEM). ^‡^Denotes significant differences between the mean time lapses between these four doses compared to the control (H0M0). *Denotes a significant difference between the H0.5M0 and the H0.5/M0.5 group. **d** The effect of the chronic treatment with haloperidol and MDL-100,907 on the induction of VCM/2min (mean ± SEM). ^‡^Denotes a significant increase in the number of the VCM compared to vehicle-treated rats.

In control animals (H0M0, neither dizocilpine nor haloperidol/MDL-100,907 treatment) the PPI (both auditory stimuli volumes combined) was, in average, 62% (Fig. 3a). As expected, dizocilpine disrupted PPI in control rats, diminishing it, in average, to 31% (*p* < 0.01). Given that the hypothesis under evaluation concerned the ability of the various combinations of haloperidol and MDL-100,907 to reverse the effect of dizocilpine on PPI, a MANOVA was performed only on the dizocilpine-treated rats, using the PPI (%) responses after 80 (Fig. 3a) and 85 dB (Fig. 3b) as dependent variables and the haloperidol and MDL-100,907 doses as factors. When added to the various doses of haloperidol, a significant effect of MDL-100,907 treatment (*p* < 0.05) on dizocilpine-induced PPI disruption was observed, at least at the lowest doses of haloperidol (0.1 and 0.25 mg/kg/day). In addition, a significant interaction between the haloperidol and MDL-100,907 factors was observed (*p* < 0.05). Post hoc analysis using a protected Fischer’s least significant differences (LSD) test failed to demonstrate signification differences between any of the individual haloperidol and MDL-100,907 dosage combinations and the control group.

### Haloperidol-induced catalepsy reversal by MDL-100,907

Chronic haloperidol doses up to 0.25 mg/kg/day, alone or in combination with 1 mg/kg/day MDL-100,907, had no effect on catalepsy (Fig. 3c). In contrast, haloperidol doses of 0.5 mg/kg/day and 1 mg/kg/day induced a strong catalepsy, measured as the time elapsed between the placement of the animal on the grid and their first paw movements (111.4 ± 52.5 and 137.8 ± 79.1 s to first movement, respectively, Fig. 3c). This difference was statistically significant as revealed by a Kruskal–Wallis test, *p* < 0.001 and post hoc Mann–Whitney tests *p* < 0.05). Adding a 5-HT_2A_R antagonism alleviated the cataleptic effect of haloperidol at 0.5 mg/kg/day (40.3 ± 26.9 s, *p* < 0.05) but not at 1 mg/kg/day (122.1 ± 92.2 s, *p* > 0.05).

### Haloperidol-induced vacuous VCM

A chronic treatment with doses of haloperidol of 0.5 and 1 mg/kg/day induced a significant increase in the number of the VCM (18.7 ± 5.8 and 13.3 ± 8.2, respectively) when compared to vehicle-treated rats (2.4 ± 2.11, Kruskal–Wallis, *p* < 0.001 and Mann–Whitney test for post hoc comparisons, *p* < 0.05). Lower haloperidol doses of 0.1 and 0.25 mg/kg/day induced no VCM. On the other hand, MDL-100,907 treatment had no effect on this phenomenon, i.e. did not manage to alleviate the haloperidol-induced VCM syndrome (Fig. 3d).

### Alteration in D_2/3_R and 5-HT_2A_R binding after chronic antagonism

**Fig. 4:**
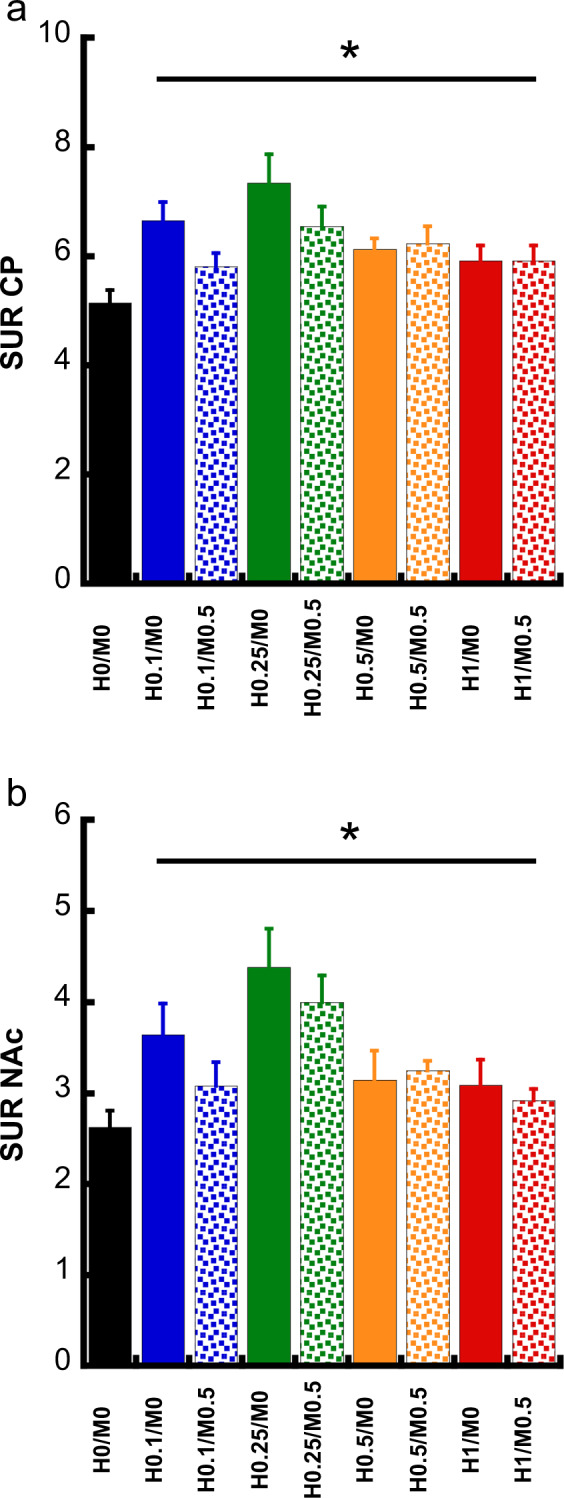
D_2/3_ binding alterations after chronic haloperidol and MDL-100,907 treatment. Receptor quantification was performed in the CP (**a**) and NAc (**b**) (mean ± SD), one week after the end of the treatment period. *All groups present a significant increase compared to the controls (H0/M0).

**Fig. 5:**
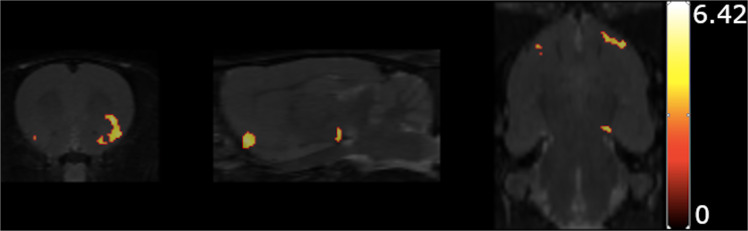
Results of the voxel-wise comparison of the 5-HT_2A_R binding between the rats of the control group (H0/M0) and the rats treated with haloperidol at 0.25 mg/kg/day.

Figure 4a and b show the effect of chronic treatment with the various doses of haloperidol and MDL-100,907 on D_2/3_R binding in the CP and the Nucleus Accumbens (NAc), respectively. All doses of haloperidol induced a significant up-regulation of D_2/3_ binding in both regions compared to the vehicle-treated groups, as measured with [^123^I]IBZM, one week after the end of the treatment period (*p* < 0.001 for the effect of haloperidol using a two-way MANOVA and significant post hoc tests of all doses against the vehicle-treated group). The addition of MDL-100,907 had no effect on this haloperidol-induced D_2/3_ up-regulation in either the CP or the NAc (Fig. 4a, b). For the analysis of D_2/3_R-binding alterations, only VOI-wise analysis was performed, given that the distribution of [^123^I]IBZM binding is restricted in the NAc and the CP. Statistical comparison of the effect of the different treatment doses on [^125^I]R91150 binding was performed at the voxel level using SPM. Only the haloperidol dose of 0.25 mg/kg/day has a significant effect on 5-HT_2A_R binding (Fig. 5) on a collection of frontal cerebral voxels, encompassing parts of the left orbitofrontal, piriform, insular and olfactory cortex, the right piriform and olfactory cortex as well as the left ventral tegmental area (VTA).

## Discussion

### Strengths of the in-vivo imaging approach and design of the study

This study described a thorough evaluation of the impact of 5-HT_2A_R antagonism on multiple aspects of the efficacy and side effect profile of haloperidol. It presents a certain number of strengths regarding its design, the variety of outcome measures and the methods to evaluate these outcome measures. The major strength of the present study is, to our view, the carefully chosen doses of haloperidol for the chronic treatment that were both representative of what has been employed in the literature and clinically relevant. Indeed, we employed doses ranging from relatively low (0.1 mg/kg/day) to particularly high (1 mg/kg/day). The 0.1 mg/kg/day dose is particularly interesting as it produces an occupancy of around 45–60% of the D_2/3_ receptors in the striatum, i.e. subtherapeutic or at the lowest end of the occupancy window that is considered optimal^1,7^. This occupancy was confirmed both ex vivo and in vivο. The 1 mg/kg/day dose was included in this study to allow a direct comparison with the majority of previous studies in the field. As discussed in the subsequent sections of this paper, the use of a 1 mg/kg/day dose of haloperidol in the literature (which has been criticized as unreasonably high^7^) may “conceal” any ameliorative effect of co-administered agents, such as the MDL-100,907. On the contrary, the use of a 0.5 mg/kg/day dose almost saturates striatal D_2/3_R (Fig. 2), induces clinically relevant side effects (e.g. catalepsy and VCM) and allows to demonstrate potential ameliorative effects of MDL-100,907, that were previously unappreciated in the literature.

A chronic treatment scheme with the D_2/3_R and 5-HT_2A_R antagonists was chosen. This is probably more clinically relevant for the evaluation of the effects of these antagonists firstly because antipsychotic agents are almost always employed chronically in patients. Secondly, the administration of these antagonists using osmotic minipumps and not via daily injections induces a stable occupancy of the receptors over time^31^, resembling the temporal pattern of occupancy in patients.

### 5-HT_2A_R antagonism partially alleviates haloperidol-induced catalepsy but has no effect on VCM

The present study confirms and extends the existing literature on the effect of a 5-HT_2A_R antagonism on the behavioral and neurochemical alterations induced by a D_2/3_R-specific antagonist. An interesting finding of our study concerns the effect of MDL-100,907 on haloperidol-induced catalepsy. As expected, a high occupancy of the striatal D_2/3_R induces this acute extrapyramidal symptom. Rats treated with the two higher haloperidol doses (0.5 and 1 mg/kg/day) showed a striatal occupancy >80% and presented a strong catalepsy, which confirms current literature^8–12^. In accordance with this literature, 5-HT_2A_R antagonism failed to counteract the cataleptic effect of the highest dose of haloperidol (1 mg/kg/day). Interestingly, 5-HT_2A_R antagonism managed to significantly reduce the cataleptic effect of the 0.5 mg/kg/day dose of haloperidol, a finding that, to our knowledge, has never been reported before. Indeed, previous studies which evaluated the effect of MDL-100,907 on chronic haloperidol-induced catalepsy only employed high doses of haloperidol (1 mg/kg/day or higher)^8,10^. Regarding acute treatment regimes, Creed-Carson et al.^10^ employed a single subcutaneous 0.5 mg/kg dose of haloperidol. The resulting catalepsy was not reversed by a 0.5 mg/kg dose of MDL-100,907 (the exact same dose as in the present study). Similar results were observed with an acute administration of 0.63 mg/kg of haloperidol^32^ and MDL-100,907 at 0.1 mg/kg. However, this apparent discrepancy might be explained by the differential effects of an acutely vs. chronically administered dose of haloperidol and by the lower dose of MDL-100,907 employed in the latter study. Indeed, it is probable that the duration of treatment with an antagonist has an impact on the relationship between its dose and the resulting catalepsy: an acute dose of haloperidol at 0.25 mg/kg induces catalepsy, while the same dose administered chronically does not^33^. In addition, in studies comparing a continuous vs. once daily administration of haloperidol via subcutaneous injections, it was demonstrated that the same dose of haloperidol, when administered once daily, produces steep peaks in occupancy that are considerably higher than the occupancy that is achieved with a continuous treatment via osmotic minipumps^23,31^. Regarding other possible pharmacological targets against antipsychotic-induced catalepsy, the 5-HT_2C_ receptor could be another candidate receptor that could be related to atypicality. Indeed, 5-HT_2C_ antagonism is a common characteristic of atypical antipsychotic agents^34,35^. A chronically administered dose of haloperidol at 1 mg/kg/day produces a catalepsy that may be reversed by a 5-HT_2C_ antagonism^9,10,35^ and 5-HT_2C_ antagonism may also reverse raclopride (a highly selective D_2/3_ antagonist)-induced catalepsy^36,37^. In the Creed-Carson study described above, 5-HT_2C_R antagonism was even superior to 5-HT_2A_R antagonism in reversing catalepsy induced by a single 0.5 mg/kg dose of haloperidol^10^. In a recent meta-regression study, 5-HT_2C_R affinity of antipsychotic agents was inversely associated to the risk of EPS in clinical studies^34^. In light of these findings, a synergistic modulation of the nigrostriatal system by both 5-HT_2A_R and 5-HT_2C_R may be hypothesized^38^. A 5-HT_2A_R antagonism may only be effective to prevent catalepsy within a limited range of D_2/3_R blockade^39,40^. Overall, these results suggest that a 5-HT_2A_R antagonism could—at least partially—mediate the clinically observed lower prevalence of acute extrapyramidal symptoms with atypical antipsychotic agents^3^.

The second aspect of motor side effects evaluated in this study was haloperidol-induced VCM. In accordance with the literature^11,22,23^, our results demonstrate that a high occupancy of the striatal D_2/3_R (induced by haloperidol doses of 0.5 and 1 mg/kg/day) is associated with an induction of VCM. A 5-HT_2A_R antagonism failed to alleviate this side effect of haloperidol (both at 0.5 and 1 mg/kg/day). This finding is also in accordance with and extends the existing literature that, so far, has only evaluated the effect of 5-HT_2A_R antagonism on the VCM induced by the highest dose of haloperidol (1 mg/kg/day). Here, we extend this finding for a lower, but still supratherapeutic, dose of haloperidol (0.5 mg/kg/day). Interestingly, the effect of 5-HT_2A_R antagonism not only lacked any preventive effect on the VCM but was even associated with a tendency to increase haloperidol-induced VCM (Fig. 3d, not reaching significance). Consequently, 5-HT_2A_R antagonism is probably not implicated in the clinically and experimentally observed lower prevalence of VCM in animals treated with atypical vs. typical antipsychotics^41–46^ and other mechanisms might mediate this phenomenon. In this respect, 5-HT_2C_R antagonism, which is a common feature of many atypical antipsychotics, has been proposed to play a role in the reduction of VCM in chronic haloperidol-treated rodents. One study in particular directly compared the effects of a selective 5-HT_2C_ and 5-HT_2A_R antagonism in reversing haloperidol-induced VCM and found a superior efficacy of the former treatment^10^. Moreover, another study suggested a mechanistic link between this receptor and VCM^47^. 5-HT_2C_R might thus be a more valid target of research for the prevention of antipsychotic-induced tardive dyskinesia. Finally, 5-HT_2C_ antagonism, given its role in the regulation of dopaminergic neurotransmission, could be associated to properties of atypical antipsychotic drugs beyond motor side effects^35,48,49^.

### 5-HT_2A_R antagonism alters the dizocilpine-disruption of the PPI

In the present study, our hypothesis was that adding a chronic antagonism at the 5-HT_2A_R to a chronic haloperidol treatment would allow to reverse the PPI-disruptive effect of dizocilpine. In this test, atypical antipsychotics have been found effective^50–55^, while a haloperidol-only treatment has consistently been found ineffective^51,56^ (with only one study, to our knowledge, showing efficacy of a 14-day haloperidol treatment at 1 mg/kg/day in mice^54^). Given that a 5-HT_2A_R antagonism alone has given positive results in one study^18^, one might consider that a chronic MDL-100,907 treatment could render the haloperidol treatment capable of reversing the effect of dizocilpine and thus provide evidence that a 5-HT_2A_R antagonism could be the substrate of the superiority of atypical agents over typical ones in this experimental paradigm. Our results showed a positive effect of MDL-100,907 on PPI, likely dependent on the concurrently administered dose of haloperidol. The absence of significant results in the post hoc tests may be explained by a lack of the necessary statistical power to clearly demonstrate significant differences in group-wise comparisons. Overall, the results of the PPI experiments provide further argument in favor of the efficacy of a 5-HT_2A_R antagonism in the reversal of dizocilpine-disruption of the PPI but further research is needed to confirm this result.

### 5-HT_2A_R antagonism fails to reverse the haloperidol-induced D_2/3_ upregulation

At a neurochemical level, chronic D_2/3_R antagonism by haloperidol led to a significant increase in the D_2/3_R binding in the CP and the NAc, an effect observed over the whole range of haloperidol doses. This is in accordance with the literature, in which a chronic D_2/3_R antagonism has been shown to upregulate striatal D_2/3_R^22,23,57–61^. The literature also suggests that this D_2/3_ upregulation is present to a lesser extent, if at all, with atypical antipsychotics, notably clozapine^57,62–64^. Given the affinity for the 5-HT_2A_R of the majority of atypical agents that were evaluated in these studies, it was proposed that a 5-HT_2A_R antagonism could prevent this D_2/3_R upregulation. To our knowledge, no study so far has assessed the effect of a 5-HT_2A_R antagonism on this phenomenon to directly test this hypothesis. In the present study, the co-administration of MDL-100,907 with any of the doses of haloperidol failed to significantly prevent haloperidol-induced D_2/3_R upregulation, suggesting that 5-HT_2A_R antagonism may not be implicated in the lack of D_2/3_R upregulation with atypical antipsychotics and other receptors could account for this phenomenon^22,23,57–61^.

It is also hypothesized that haloperidol-induced D_2/3_R upregulation is implicated in the occurrence of VCM^22^. The results obtained here suggest that D_2/3_R upregulation is probably not a sufficient condition for the induction of VCM, as the animals treated with the 0.1 and the 0.25 mg/kg/day doses presented a D_2/3_R upregulation without VCM. These results challenge the hypothesized causal link between D_2/3_R upregulation and VCM induction and emphasize the need to conduct in-depth studies of these two phenomena.

### Haloperidol at moderate doses upregulates the 5-HT_2A_R in frontal cortical areas and the VTA

A surprising finding of the present study was the increase in 5-HT_2A_R binding in frontal cortical areas and in the VTA, induced by a moderate dose of haloperidol (0.25 mg/kg/day). This effect was unaltered by 5-HT_2A_R antagonism. In fact, chronic 5-HT_2A_R antagonism was not associated with any change in either D_2/3_R or 5-HT_2A_R availabilities. This is a previously unappreciated finding, given that the literature so far has not assessed the effect of such a moderate dose of haloperidol on 5-HT_2A_R binding. Charron et al.^65^ showed that haloperidol, at 0.5 mg/kg/day decreases [^3^H]ketanserin binding in the frontal cortex and increases it in the striatum. However, this radiotracer also binds to 5-HT_2C_R, rendering the interpretation of these findings difficult. The downregulation of 5-HT_2A_R^66–74^, shared by atypical but not typical agents, was hypothesized as one of the substrates of atypicality, but no conclusive evidence linking it to the efficacy and side effect profile of atypical agents has been reported so far. The present study, given the absence of any 5-HT_2A_R antagonist properties of haloperidol, points to an indirect modulation of 5-HT_2A_R binding. One explanation for this could involve an alteration of serotonin release. If serotonin release is diminished, this transmitter would compete less with the [^125^I]R91150 radiotracer for binding to the 5-HT_2A_R, leading to an increase in radiotracer binding. Indeed, there is evidence that the dopaminergic system, via the D_2_ receptor, may alter serotonin transmission^75–80^. Previous studies showed that haloperidol treatment leads to a reduction in the concentration of a serotonin metabolite^81^ and serotonin itself in the brain^82^. Another possible hypothesis would be to attribute this haloperidol-induced change of 5-HT_2A_R binding to alterations in D_2_/5-HT_2A_ heteromers. Albizu et al.^83^ found that heteromers of D_2_R and 5-HT_2A_R produce allosteric modulations of the latter receptor via D_2_-mediated mechanisms and alter its affinity for 5-HT_2A_R-binding radioligands. Finally, to explain the differential effect of moderate vs. high doses of haloperidol on 5-HT_2A_R binding, one could hypothesize that low doses of haloperidol may preferentially act on D_2_ autoreceptors, while higher doses act both on auto- and hetero-receptors^84^.

Regarding the possible functional implications of this finding, previous data has highlighted a differential interaction between 5-HT_2A_R-mediated and D_2/3_R-mediated effects depending on the level of D_2/3_R occupancy. Indeed, Liegeois et al.^40^ and Bonaccorso et al.^48^ showed that in vivo 5-HT_2A_R blockade with MDL-100,907 potentiated the dopamine-releasing effect of haloperidol in the rat medial prefrontal cortex, but only when haloperidol was administered at a dose lower or equal to 0.1 mg/kg. This could possibly be explained by our finding that a similar dose of haloperidol alters 5-HT_2A_R binding in rat frontal cortical areas. From a functional perspective, this could provide a mechanism through which a relatively low D_2/3_R occupancy combined with a 5-HT_2A_R occupancy mediates the effect of atypical antipsychotics by relatively preserving dopaminergic transmission in the frontal cortex while potently blocking it in the mesolimbic system^5^. The marked expression of 5-HT_2A_R in prefrontal cortical neurons that project to the NAc and the VTA suggests that the haloperidol-induced 5-HT_2A_R upregulation might be relevant for the regulation of dopaminergic neurotransmission by antipsychotic medications^85,86^. Interestingly, in the present study, the dose of 0.25 mg/kg/day of haloperidol, is the dose at which the impact of 5-HT_2A_R antagonism shows the highest tendency towards a reversal of dizocilpine-disruption of PPI. 5-HT_2A_R upregulation could be one of the mechanisms through which 5-HT_2A_R antagonism at this particular dose of haloperidol (0.25 mg/kg/day) may potentiate its efficacy on PPI. 5-HT_2A_R is also implicated in cognitive processes that may be deficient in patients suffering from schizophrenia and/or who are treated with antipsychotic medication. This could be relevant to the haloperidol-induced 5-HT_2A_R upregulation^87^. Nevertheless, further experiments, e.g. a cell-specific manipulation of 5-HT_2A_R signaling, are needed to directly interrogate the molecular underpinnings of the D_2/3_R-occupancy-dependent 5-HT_2A_R alteration in 5-HT_2A_R binding and assess its functional implications in terms of possible behavioral effects.

### Limitations of the present study

The use of a Mdr1a knock-out strain may be considered a limitation. We used this strain to be able to use [^125^I]R91150 for the in-vivo imaging of 5-HT_2A_R^14,88,89^. The use of Mdr1a knock-out rats probably does not bias the behavioral and neurochemical responses to the chronic treatment with haloperidol and MDL-100,907, given that: (1) Mdr1a knock-out and wild-type rats present identical D_2/3_R and 5-HT_2A_R binding in the brain, as confirmed by ex-vivo autoradiography which is possible with [^125^I]R91150 given the highest sensitivity of the autoradiography when compared to in-vivo SPECT, even in wild-type rats^14,89^, (2) the dose-occupancy curve of haloperidol measured here in Mdr1a knock-out is similar to that previously reported in wild-type rats^12,90^, (3) the behavioral responses to haloperidol and dizocilpine were highly comparable to those observed in previous studies, notably the correspondence of the D_2/3_R occupancy by haloperidol and the induction of side effects^8–12,22^.

## Conclusion

In conclusion, we provide evidence for the involvement of 5-HT_2A_R antagonism in the alleviation of catalepsy induced by haloperidol, an effect that is dose-dependent. Similarly, evidence is provided for an involvement of 5-HT_2A_R antagonism on the reversal of dizocilpine-disruption of PPI. 5-HT_2A_R antagonism failed to prevent the upregulation of D_2/3_R that is induced by chronic haloperidol treatment, as well as the induction of VCM by high doses of this typical antipsychotic agent. A previously unappreciated dose-dependent effect of moderate doses of haloperidol on the in-vivo frontal cortical 5-HT_2A_R binding has also been observed. The present work points to an involvement of a 5-HT_2A_R antagonism in the modification of some aspects of the efficacy and side effect profile of haloperidol, suggesting that, at least partially, 5-HT_2A_R antagonism might be associated with atypicality. Based on the results of this study however, the role of the 5-HT_2A_R antagonism as the sole (or even the major) determinant of antipsychotic atypicality can probably be rejected. The need to carefully choose clinically relevant antipsychotic doses (i.e. a dose of 0.5 mg/kg/day and not 1 mg/kg/day) and to further investigate the role of neurochemical changes induced by chronic antipsychotic treatment in the search for causal relationships with its clinical effect is warranted.

## Supplementary information

Supplemental materials and methods
