# Supplementary material for: Effect of 5-HT2A receptor antagonism on levels of D2/3 receptor occupancy and adverse behavioral side-effects induced by haloperidol: a SPECT imaging study in the rat
Source: Transl Psychiatry. 2021 Jan 14;11:51. doi: 10.1038/s41398-020-01179-5 (PMC7809418; doi:10.1038/s41398-020-01179-5)
Supplement: Supplementary file 1 — Supplemental materials and methods [file 41398_2020_1179_MOESM1_ESM.docx]

**Supplemental materials and methods**

*Behavioral testing*

*Dizocilpine-disrupted prepulse inhibition (PPI) of the startle reflex*

In rodents, exposure to a strong acoustic stimulus provokes a startle response. If this strong stimulus is preceded by a milder acoustic stimulus, then the response of the animal to the startle-eliciting stimulus is attenuated and this phenomenon is termed prepulse inhibition (PPI) of the startle. It is disrupted by a dizocilpine (MK801) pretreatment^1-3^. Antipsychotic agents reverse this disruption and this property is considered as a proxy of their efficacy against psychotic symptoms^1-3^.

Between 16 and 19 days following implantation of the osmotic minipumps, startle reactivity was measured in sound-attenuating startle chambers (TSE Systems, Bad Homburg, Germany), which include enclosures (22.5x8x8.5cm) equipped with loudspeakers and a piezoelectric accelerometer that allow to deliver tone pulses and to measure animal startle responses, respectively. The first two days consist of habituation sessions (10 min and 30 min of 70dB background noise on day 1 and day 2, respectively). On day 3, PPI was measured (immediately after an i.p. injection of saline) as follows: after a 10 min acclimation period (70dB), the rat received, in a random fashion, 24 trials with a pulse-alone stimulus (120 dB, 40 ms), 12 trials with no stimulus (70-dB 200 ms), two types (3 × 12) of prepulse-and-pulse trials which include a 20-ms prepulse (80 and 85-dB) followed 100 ms later by a 120-dB pulse stimulus, as described previously^4^. On day 4, PPI was measured as on day 3, using dizocilpine (0.15mg/kg) instead of saline as pretreatment. The amplitude of startle responses was recorded in all trials. The magnitude of PPI was calculated as a percent inhibition of the startle amplitude in the pulse-alone trial^3, 5^.

*Catalepsy*

Catalepsy is indicative of the potential of a pharmacological agent to induce extrapyramidal symptoms^6, 7^. At 25 days following minipump implantation, catalepsy was assessed using a steel grid floor that was inclined at 60°. Rats were placed on the grid and the time elapsed without any front paw movements was recorded for a maximum of 3 min and used to estimate catalepsy^8^.

*Ex vivo receptor binding measurements and in vivo imaging*

*Radiotracer preparation*

Preparation of [^123^I]IBZM and [^125^I]R91150 was performed as described previously by our group^9-11^. All chemicals for radiotracer preparation were purchased from Sigma-Aldrich (Buchs, Switzerland) unless otherwise specified. ^123^I and ^125^I radioiodine were purchased from Perkin Elmer (Basel, Switzerland). [^123^I]IBZM was obtained by incubation, for 15 min at 68°C, of a mixture containing 5 μl of BZM precursor (ABX, Germany, 24 nmol/ μl in ethanol), 2 μl of glacial acetic acid, 1 μl of 30% H_2_O_2_ and 10 mCi of carrier-free ^123^I sodium iodide in 0.05 M NaOH. The radiotracer was isolated by a linear gradient HPLC run (from 5% acetonitrile, ACN, to 95% ACN, 10 mM H_3_PO_4_, in 10 min). HPLC was equipped with a reverse-phase column (Phenomenex Bonclone C18, Phenomenex, Schlieren, Switzerland) and radiotracer was eluted at a flow of 3 ml/min. Fractions containing [^123^I]IBZM were diluted in water and loaded on a Sep-Pak cartridge (Sep-Pak C18, Waters, Switzerland). [^123^I]IBZM was eluted with 0.5 ml of 95% ACN, 10 mM H_3_PO_4_ and concentrated using a rotary evaporator, and the final product was diluted in saline prior to animal administration.

R91150 precursor preparation was described elsewhere^12^. For radiolabelling, 300 μg of R91150 precursor in 3 μL ethanol was mixed with 3 μL of glacial acetic acid, 15 μL of carrier-free ^125^I sodium iodide (10 mCi) in 0.05 M NaOH, and 3 μL of 30% H_2_O_2_. [^125^I]R91150 was isolated by an isocratic HPLC run (ACN/water 50/50, 10 mM acetic acid buffer pH 5) with a reversed-phase column (Bondclone C18 10 μm 300 X 7.8 mm, Phenomenex, Schlieren, Switzerland) at a flow rate of 3 mL/min.

*Ex vivo estimation of receptor occupancy by haloperidol and MDL-100,907*

In the *ex vivo* dose-occupancy curve estimations, rats were administered with [^123^I]IBZM and [^125^I]R91150 to concurrently measure D_2/3_ and 5-HT2A receptor occupancy, respectively. At 28 days of treatment, rats were anesthetized using isoflurane anaesthesia (4% for induction, 2.5% for maintenance) and injected with 6.48±0.34 MBq of [^123^I]IBZM or 6.98±0.98 MBq of [^123^I]R91150 (depending on the administered antagonist, haloperidol or MDL-100,907, respectively). At 120 min post-injection, rats were euthanized by decapitation, their brain removed, and their striatum, frontal cortex and cerebellum dissected and weighed. Radioactivity in the dissected brain regions was immediately measured in an automated gamma counting system (expressed in KBq/g of tissue weight) for the radiotracer labelled with ^123^I. Radioactivity was decay-corrected to the time of the brain dissection.

For the *ex vivo* study, the standardized uptake ratio (SUR) for each radiotracer in the striatum and the frontal cortex was measured using the radioactivity measured in the gamma counting system as follows: SUR=(radioactivity in the target-region)/(radioactivity in the cerebellum) – 1. The % occupancy (O) of the D_2/3_ and the 5-HT_2A_ receptors from their respective antagonists was estimated using the following formula: O (%)=(1-SUR/SUR_CON_)*100, where SUR corresponds to the value obtained from an individual study in which a dose of antagonist was employed, while SUR_CON_ corresponds to the average value obtained from the control animals in which no antagonist was administered.

*In vivo imaging experiments*

Dual-radiotracer SPECT imaging^11^ was performed in the context of the main *in vivo* study described in this paper to assess the level of D_2/3_ and 5-HT_2A_ occupancy by haloperidol and MDL-100,907 and the binding of D_2/3_ and 5-HT_2A_ receptors after chronic treatment with these agents. *In vivo* dual radiotracer SPECT was performed as described previously^11^. At the end of the 28-day treatment period, the first dual-radiotracer SPECT scan was performed, to measure the occupancy of the D_2/3_ and the 5-HT_2A_ receptor by their respective antagonists. One week later, an exactly similar dual-radiotracer SPECT scan was performed to index the density of the D_2/3_ and the 5-HT_2A_ receptors. A polyethylene catheter (22G) was inserted in the tail vein for radiotracer injection, at a volume of 0.6 ml. Rats were simultaneously injected with a mixture of [^123^I]IBZM (32.7±8.2 MBq) and [^125^I]R91150 (26.9±6 MBq) over 30 sec. At 80 minutes post-radiotracer administration, rats were anesthetized using isoflurane (4% for induction and 2.5% for maintenance) and the scan was initiated in a U-SCAN-II SPECT camera (MiLabs, Utrecht, Netherlands) (using 4 frames of 10-min each). The choice of this timing for the SPECT scans relies on previous work from our group and allows the quantification of both radiotracers’ binding: indeed, the SUR of [^123^I]IBZM is estimated over the static images corresponding to 80-110 min after the injection of the radiotracer^10^, while the SUR of [^125^I]R91150 at 100-120 min^9^. Body temperature was maintained at 37±1 **°**C by means of a thermostatically controlled heating blanket.

SPECT image reconstruction was performed using a pixel ordered subsets expectation maximization (P-OSEM, 0.4 mm voxels, 4 iterations, 6 subsets) algorithm using the MiLabs image reconstruction software. Reconstruction of dynamic SPECT images was performed using the radioactivity measured at each radioisotope’s principal energy spectrum, that is at 143,1-179,9 keV for ^123^I and at 15-45 keV for ^125^I. Radioactive decay correction was performed while correction for attenuation or scatter was not.

*Separation of the two distinct images from the dual-radiotracer SPECT scan*

The co-injection of [^123^I]IBZM with [^125^I]R91150 induces a contamination of the [^125^I]R91150 image. ^123^I emits radioactivity principally at the 143,1-179,9 keV energy spectrum but also at a secondary energy spectrum, which is exactly the spectrum of ^125^I (15-45 keV). To correct for this contamination, we employed a method described previously by our group^11^. Briefly, the secondary emission of ^123^I is directly related to the principal one (34%). Contaminated [^125^I]R91150 images were thus corrected by subtracting 34% of the radioactivity measured in the [^123^I]IBZM images at the 143,1-179,9 keV energy spectrum.

*Standardized uptake ratio (SUR) estimation in the in vivo imaging experiments*

For the *in vivo* study, SPECT images were processed using PMOD software (version 3.9, PMOD Technologies Ltd, Zurich, Switzerland). For each rat, static [^125^I]R91150 SPECT images were spatially normalized on a [^125^I]R91150 template-image, as previously described^11^, and filtered with a Gaussian filter of 0.6m^3^ FWHM. The resulting transformation matrix was applied to the corresponding [^123^I]IBZM images for each rat. A volume-of-interest (VOI) template (including 57 VOIs), incorporated in PMOD^13^ was used to extract the radioactivity from each brain VOI and the cerebellum (CER), which was used as reference region. SUR values were estimated as follows: (Radioactivity in the target VOI)/(Radioactivity in CER)-1. The SUR values of the first dual-radiotracer SPECT scan, which was performed on the 28^th^ day of the treatment period, were used to estimate the occupancy of the receptor by their respective antagonists. For estimation of D_2/3_ receptor occupancies using *in vivo* imaging with [^123^I]IBZM, a 0.55 value was subtracted from the SUR and SUR_CON_ values. This value corresponds to the difference in the non-displaceable binding between the striatum (target region) and the cerebellum (reference region) for this radiotracer, as described in a previous paper from our group^10^.. The SUR values of the second SPECT scan for each rat (which is performed 7 days after the end of the treatment period) are direct indexes of the density of the respective receptor populations. For the [^125^I]R91150 images of the second SPECT scans, SUR was also estimated at the voxel level using the same formula.

**References**

1. Swerdlow NR, Weber M, Qu Y, Light GA, Braff DL. Realistic expectations of prepulse inhibition in translational models for schizophrenia research. *Psychopharmacology* 2008; **199**(3)**:** 331-388.

2. Wadenberg MG, Sills TL, Fletcher PJ, Kapur S. Antipsychoticlike effects of amoxapine, without catalepsy, using the prepulse inhibition of the acoustic startle reflex test in rats. *Biological psychiatry* 2000; **47**(7)**:** 670-676.

3. Varty GB, Bakshi VP, Geyer MA. M100907, a serotonin 5-HT2A receptor antagonist and putative antipsychotic, blocks dizocilpine-induced prepulse inhibition deficits in Sprague-Dawley and Wistar rats. *Neuropsychopharmacology : official publication of the American College of Neuropsychopharmacology* 1999; **20**(4)**:** 311-321.

4. Tournier BB, Ginovart N. Repeated but not acute treatment with (9)-tetrahydrocannabinol disrupts prepulse inhibition of the acoustic startle: reversal by the dopamine D(2)/(3) receptor antagonist haloperidol. *European neuropsychopharmacology : the journal of the European College of Neuropsychopharmacology* 2014; **24**(8)**:** 1415-1423.

5. Martinez ZA, Oostwegel J, Geyer MA, Ellison GD, Swerdlow NR. "Early" and "late" effects of sustained haloperidol on apomorphine- and phencyclidine-induced sensorimotor gating deficits. *Neuropsychopharmacology : official publication of the American College of Neuropsychopharmacology* 2000; **23**(5)**:** 517-527.

6. Kapur S, Wadenberg ML, Remington G. Are animal studies of antipsychotics appropriately dosed? Lessons from the bedside to the bench. *Canadian journal of psychiatry Revue canadienne de psychiatrie* 2000; **45**(3)**:** 241-246.

7. Ginovart N, Kapur S. Role of dopamine D(2) receptors for antipsychotic activity. *Handbook of experimental pharmacology* 2012; (212)**:** 27-52.

8. Gobira PH, Ropke J, Aguiar DC, Crippa JA, Moreira FA. Animal models for predicting the efficacy and side effects of antipsychotic drugs. *Rev Bras Psiquiatr* 2013; **35 Suppl 2:** S132-139.

9. Dumas N *et al.* In Vivo Quantification of 5-HT2A Brain Receptors in Mdr1a KO Rats with 123I-R91150 Single-Photon Emission Computed Tomography. *Molecular imaging* 2015; **14**.

10. Tsartsalis S *et al.* A single-scan protocol for absolute D2/3 receptor quantification with [123I]IBZM SPECT. *NeuroImage* 2017; **147:** 461-472.

11. Tsartsalis S *et al.* Dual-radiotracer translational SPECT neuroimaging. Comparison of three methods for the simultaneous brain imaging of D2/3 and 5-HT2A receptors. *Neuroimage* 2018; **176:** 528-540.

12. Dumas N *et al.* Small-animal single-photon emission computed tomographic imaging of the brain serotoninergic systems in wild-type and mdr1a knockout rats. *Molecular imaging* 2014; **13**(1).

13. Schiffer WK *et al.* Serial microPET measures of the metabolic reaction to a microdialysis probe implant. *J Neurosci Methods* 2006; **155**(2)**:** 272-284.
